# Recent Legal Decisions

**Published:** 1913-06-28

**Authors:** 


### RECENT LEGAL DECISIONS.

We recently noticed in this column the case of The Scottish National Insurance Commissioners *v.* The Edinburgh Royal Infirmary, in which it was held that the infirmary surgeons were outwith the scope of the Insurance Act in respect that there was no contract of service between them and the directors of the institution. On similar lines is the judgment just issued in The Directors of the House of Refuge for the Destitute *v.* The Scottish Insurance Commissioners, 1913, 1 S.L.T. 461, which finds an inmate of a House of Refuge for the Destitute who did menial work in the house and received a small gratuity weekly was not employed under a contract of service in the meaning of the National Insurance Act. "The weekly money payment is not wages or part of wages for services rendered under a contract. It is a gratuity or bonus as a spur to good conduct given or not given at the governors' pleasure."

The Dentists Act, 1878, does not, it has been held, preclude an unqualified person from fitting artificial teeth nor even from extracting natural ones, which is a near approximation to dental surgery, but it does preclude him from using any description implying that he is a dental surgeon. In Robertson *v.* Hawkins, 29 T.L.R. 32, the appellant, an assistant schoolmaster under the London County Council, received a notice from the Council requiring him to obtain not later than January 12, 1912, a dental certificate, and requesting him to show the notice to the dentist as only the certificate of a registered dentist would be accepted. The appellant on December 12, 1911, took the notice to and showed it to the respondent, who carried on the practice of dentistry under the name of "Williams' Artificial Teeth Institute," but who was neither a registered dentist nor a legally qualified medical practitioner, and the appellant had his teeth extracted. The respondent did not at any time, either in writing or orally, use the title or words "dentist" or "registered dentist" or state that he was a person specially qualified to practise dentistry, but it was found that the appellant by words and actions told the respondent he required a certificate by a registered dentist, that the respondent stated to the appellant that he had granted hundreds of such certificates, and that he would give the required certificate, on January 12, 1912. No such certificate was given. On a prosecution of the respondent for having unlawfully used and taken the name or title of registered dentist the magistrate dismissed the charge. It was held, on appeal, that the respondent had committed an offence against the Dentists Act, 1878, by saying, by his words and conduct, that he was a registered dentist, and, therefore, that he should have been convicted.

An important medico-legal point which arises in connection with the Workmen's Compensation Act is whether an operation may not have the effect of altogether preventing the recovery of compensation. A very good illustration of this is the recent decision in Mutter, Howey, and Company *v.* Thomson, 50 S.L.R. 477. By an accident in the course of his employment a workman ruptured himself so that an operation was rendered necessary. At the time of the accident he was already suffering from another rupture of long standing. A double operation was performed, both hernias being operated upon, and the workman died. The medical evidence indicated that in order to operate successfully on the later hernia it was necessary to operate on the earlier one also. The Sheriff who heard the evidence found that the cause of death was heart failure due to the strain of an operation, necessitated by the accident. The accident being thus the efficient cause of the man's death the statute was found applicable, and the workman's representatives were awarded compensation. An appeal was brought, and it was argued that the death was not due to the accident at all, for, admitting that it resulted from the strain of the operation, yet the operation was a double one and, therefore, the strain had been caused, or at least very substantially contributed to, by operating on the earlier hernia. This amounted to saying that the second operation was unnecessary. Had there been evidence to prove that, compensation would certainly have been refused; but there was evidence to show that the operation necessary to cure the femoral hernia, which was the direct result of the accident, also involved, according to proper surgical treatment, dealing with the inguinal hernia which had previously existed. The Court, therefore, ruled that the death was attributable to the accident and adhered to the decision of the Court below. The case is one which medical practitioners should keep in view as a reminder that, as an operation on a workman may have legal consequences, its strict relation to the injury should be considered and noted.

